# Exogenous Klotho Extends Survival in COVID-19 Model Mice

**DOI:** 10.3390/pathogens12121404

**Published:** 2023-11-29

**Authors:** Farhang Alem, Natalia Campos-Obando, Aarthi Narayanan, Charles L. Bailey, Roman F. Macaya

**Affiliations:** 1 Biomedical Research Laboratory, George Mason University, 4400 University Dr., Fairfax, VA 22030, USA; falem@gmu.edu (F.A.); anaraya1@gmu.edu (A.N.); cbailey2@gmu.edu (C.L.B.); 2 Formerly at Caja Costarricense de Seguro Social, San José P.O. Box 10105-1000, Costa Rica; n.camposobando@erasmusmc.nl; 3 Department of Global Health and Population, Harvard T.H. Chan School of Public Health, 665 Huntington Ave., Boston, MA 02115, USA

**Keywords:** Klotho, COVID-19 pathogenesis, aging process, coronavirus, COVID-19 model mice

## Abstract

A striking feature of COVID-19 disease is the broad spectrum of risk factors associated with case severity, as well as the diversity of clinical manifestations. While no central agent has been able to explain the pathogenesis of SARS-CoV-2 infection, the factors that most robustly correlate with severity are risk factors linked to aging. Low serum levels of Klotho, an anti-aging protein, strongly correlate with the pathogenesis of the same risk factors and manifestations of conditions similar to those expressed in severe COVID-19 cases. The current manuscript presents original research on the effects of the exogenous application of Klotho, an anti-aging protein, in COVID-19 model mice. Klotho supplementation resulted in a statistically significant survival benefit in parametric and non-parametric models. Further research is required to elucidate the mechanistic role Klotho plays in COVID-19 pathogenesis as well as the possible modulation SARS-CoV-2 may have on the biological aging process.

## 1. Introduction

Infection by SARS-CoV-2 can cause a surprising diversity of clinical manifestations, ranging from a fully asymptomatic condition to severe cases with respiratory failure, acute kidney injury, and cytokine release syndrome, among other conditions [1].

Risk factors for severity are equally diverse [2]. However, risk factors related to premature human aging, especially chronic kidney disease (CKD) and acute kidney injury (AKI), are robustly associated with severity of SARS-CoV-2 infection [3,4,5,6]. Interestingly, the occurrence of AKI increases mortality risk not only in COVID-19 [7], but in other coronavirus-related diseases such as MERS and SARS [8].

To date, no unifying agent or signaling pathway has been identified that can explain the diversity of risk factors and clinical manifestations caused by SARS-CoV-2. We have identified Klotho, a protein that regulates mammalian aging [9], as a common factor that appears to play a central role in health conditions that predispose patients to severe COVID-19 outcomes as well as in health complications similar to those developed in this disease. To the best of our knowledge, this is the first time Klotho has been presented as a possible central agent in COVID-19 pathophysiology.

Interestingly, it has been postulated that HIV-positive people have low plasma levels of Klotho, which could potentially contribute to their higher cardiovascular risk factor [10].

The *Klotho* gene was discovered in 1997 in transgenic (*kl/kl*) mice that had this gene accidentally downregulated by an insertional mutation [9]. *Kl/kl* mice exhibited a syndrome that closely resembles human aging. *Kl* encodes a homonymous protein, α-Klotho, with hormonal activity that was later shown to suppress mammalian aging [11,12] and extend lifespan in mice with overexpression of *Klotho* [13].

The human *Klotho* gene, which contains five exons and four introns, encodes for the homonymous protein αKlotho, which is crucial in the aging process and in the metabolism of calcium, phosphate, and vitamin D [14]. αKlotho has functions similar in nature to a hormone, although its receptor has not yet been identified [15]. The αKlotho protein sequence is highly conserved across species, with humans and mice sharing 98% sequence identity for this protein [14]. In addition, the mouse and human *Klotho* genes encode a short form, secreted αKlotho, generated by alternative splicing. Secreted αKlotho has not been detected in rats.

The *Klotho* gene has been reported to encode two other proteins with distinct functions: βKlotho and Klotho-related protein. The former is linked to bile acid synthesis, whereas the function of the latter has not yet been clearly identified [14].

αKlotho is synthesized mainly in the renal tubular cells of the kidneys and in the brain choroid plexus [14]. Acute and chronic inflammatory states markedly downregulate *Klotho* expression, at least mediated by the inhibitory actions in its promoter by factors such as angiotensin II [16] and nuclear factor κ light chain enhancer of activated B cells (NF-κβ) [17], among others. In contrast, Sp1 is a transcription factor that upregulates *Klotho* expression [18]. Vitamin D indirectly upregulates *Klotho* transcription by binding to vitamin D responsive elements in its promoter [19]. In addition, erythropoietin, ras homolog gene family A, rapamycin, statins, fosinopril, and losartan all increase *Klotho* mRNA expression [14].

Three types of Klotho proteins have been identified in humans: the full-length transmembrane Klotho, soluble Klotho, and secreted Klotho [14].

Klotho exerts critical roles in the following processes relevant to aging and disease:

1. FGF23- dependent phosphate, calcium and vitamin D metabolism [20].
2. Antioxidant and anti-inflammatory activities [21].
3. Prevention of chronic fibrosis [22].
4. Tumor suppressor activities [23].
5. Anti-apoptotic and antisenescence functions [24].

For such effects, Klotho is directly or indirectly involved in several molecular pathways, such as the following:

- Klotho is the obligate co-receptor for FGF23, which is an important phosphaturic hormone [20]. Consistently with this, it is considered that FGF23 phosphaturic actions are critically dependent on Klotho [14]. Moreover, it has been considered that Klotho may have FGF23-independent phosphaturic effects [25].
- Klotho and TGF-β: Klotho at least partially protects against fibrosis by preventing TGF-β binding to its receptor and initiating a cascade [22].
- Klotho and NF-κβ: Klotho inhibits NF-κβ signaling, which exerts a key role in immune and inflammatory processes mediated by B cells, T cells, macrophages, and polymorphonuclear cells [15,26].
- Klotho and Nrf2 pathway: Nfr2 is a transcription factor that controls responses to oxidative stress, and as such is an antioxidant pathway. It is activated by Klotho [21].
- Klotho and insulin-like growth factor 1 (IGF-1): the insulin/IGF-1 signaling pathway has long been linked to aging. Klotho inhibits the IGF-1/PI3K/AkT/mTOR pathway, which is a likely mechanism for its antiaging effects [13,27].
- Klotho and WNT pathway: Klotho inhibits WNT signaling via binding to several WNT ligands, such as Wnt1, Wnt3, Wnt4, and Wnt5a [28]. This is of relevance considering that excess WNT activation is associated with cell senescence, decreased stem cell survival, and kidney fibrosis [29].

Although the main inflammatory pathway in COVID-19 has been considered the Janus Kinase pathway (JAK/STAT) [30,31], whether Klotho has a role in the JAK/STAT signaling pathway requires further research and validation. However, there is another important pathway in COVID-19 inflammatory pathogenesis, namely, NF-κβ signaling, which increases the expression of multiple genes linked to host immunity, inflammation, cell proliferation, and apoptosis [32]. Several SARS-CoV-2 proteins are able to initiate this pathologic pathway [33,34]. As previously mentioned, Klotho blocks NF-κβ signaling, seemingly through the prevention of its nuclear translocation [26], although other mechanisms which interact with the NF-κβ pathway may apply as well, such as an increase in nuclear factor erythroid 2-related factor activation [15].

From a clinical point of view, low serum levels of Klotho are strongly correlated with the same health conditions that have proven to be risk factors for severity of COVID-19, especially type 2 diabetes mellitus [35], cancer [36], and chronic kidney disease [12] (Table 1).

Klotho levels drop precipitously with AKI [39], a condition associated with the worst prognosis for COVID-19 [7]. Conversely, supplementation with exogenous recombinant Klotho or *Klotho* overexpression show improved health conditions in mouse models of the lung–kidney axis [40,41], cognitive impairment [42,43], sepsis [44], and pulmonary hypertension [45]. Low-dose exogenous Klotho supplementation has been shown to enhance memory in rhesus macaques in a recent evaluation of Klotho in primates [46]. (Table 2).

In this study, mouse models of COVID-19 were used to evaluate the potential survival benefit conferred by Klotho supplementation in mice exposed to SARS-CoV-2. The frequency and mode of administration of recombinant Klotho were both evaluated. The species specificity of the Klotho that was used as well as possible sex-determined differences in outcomes were additionally investigated. To the best of our knowledge, this is the first study to demonstrate a significant survival benefit for exogenous Klotho supplementation in a COVID-19 mouse model.

## 2. Materials and Methods

### 2.1. Animal Use Disclosure

All laboratory animal studies described in this manuscript adhered to the NIH guidelines for the Care and Use of Laboratory Animals and received Institutional Animal Care and Use Committee (IACUC) approval (IACUC Protocol number 0399).

### 2.2. Materials and Methods for the First Animal Study

Fifteen female transgenic hACE2 AC70 mice (Taconic Biosciences, Germantown, MD, USA) were used for this study. Baseline temperature and weight were recorded. The mice had an average body weight of 25 g. The fifteen mice were divided in three cohorts as follows: (1) control intraperitoneal (i.p.) cohort: five female mice received i.p. injections of vehicle (only buffer) every 24 h; (2) human Klotho cohort: five female mice received i.p. injections with recombinant human Klotho (R&D Systems, Inc., Minneapolis, MN, USA) in buffer every 24 h; and (3) mouse Klotho cohort: five female mice received i.p. injections with recombinant mouse Klotho (R&D Systems, Inc., Minneapolis, MN, USA) in buffer every 24 h. The Klotho buffer had the following composition: 150 mM NaCl and 10 mM HEPES pH 7.4.

The supplementation source for mouse Klotho was Chinese Hamster Ovary cell line CHO-derived mouse Klotho protein (Ala35-Lys982, with a C-terminal 6-His tag), while the supplementation source for human Klotho was mouse myeloma cell line NS0-derived human Klotho protein (Glu34-Ser981, with a C-terminal 6-His tag). Both had a purity of more than 90%.

All mice received daily i.p. injections of 0.5 mL containing vehicle (only buffer), recombinant human Klotho, or recombinant mouse Klotho according to the following schedule: Day 0, 1, 2, 3, 4, 5, 6, 7, 8, 9. Day 0 was the day on which the mice were challenged with exposure to SARS-CoV-2 through nasal delivery. The first i.p. injection took place on Day 0 one hour prior to SARS-CoV-2 exposure. On Day -1, all mice had transponders implanted to take temperature readings. A master solution for each type of Klotho (mouse or human Klotho) in its buffer was prepared before initiating the study. This solution was aliquoted in vials or tubes in the appropriate volume such that each vial contained enough solution for the i.p. injections corresponding to all mice in each cohort each day. All vials were frozen at −80 °C degrees, and only the daily vials were thawed for the i.p. injections of each day. Each mouse in the cohorts receiving Klotho received 1.25 micrograms of recombinant Klotho protein (mouse or human) per daily i.p. injection, resulting in a dose of 0.05 mg of Klotho per kilogram of body weight. Mice were anesthetized prior to handling for each i.p. injection.

The health of each mouse was assessed on a daily basis by scoring health parameters included in the Animal Study Clinical Monitoring Chart. All mice were followed until death. Mice either succumbed between one health monitoring record and the next, or were euthanized when individual mice reached a predetermined health endpoint as specified by the mentioned health monitoring chart.

### 2.3. Materials and Methods for the Second Animal Study

Forty transgenic hACE2 AC70 mice (Taconic Biosciences, Germantown, United States) were used for this study: twenty female and twenty male mice with an average body weight of 20 g. No transponders for temperature readings were implanted into the mice in this second study in order to reduce stress and surgical intervention in the tested animals. Body weight readings were eliminated to reduce the amount of stress the mice experienced from handling during the study. The forty mice were divided into eight cohorts, as follows: (1) male control intraperitoneal (i.p.) cohort: five male mice with vehicle (only buffer) application through i.p. injection every 12 h; (2) female control i.p. cohort: five female mice with vehicle (only buffer) application through i.p. injection every 12 h; (3) male control minipump cohort: five male mice with vehicle (only buffer) infusion through osmotic minipump; (4) female control minipump cohort: five female mice with vehicle (only buffer) infusion through osmotic minipump; (5) male Klotho i.p. application cohort: five male mice with recombinant mouse Klotho application through i.p. injection every 12 h; (6) female Klotho i.p. application cohort: five female mice with recombinant mouse Klotho application through i.p. injection every 12 h; (7) male Klotho minipump application cohort: five male mice with recombinant mouse Klotho infusion through osmotic minipump; and (8) female Klotho minipump application cohort: five female mice with recombinant mouse Klotho infusion through osmotic minipump. Recombinant mouse Klotho protein was acquired from R&D Systems, Inc.

### 2.4. Mice with Osmotic Minipump Implants

On day -2, all mice from cohorts 3, 4, 7, and 8 underwent subcutaneous implantation of the Alzet 1002 osmotic minipump (Durect Corporation, ALZET Osmotic Pumps, Cupertino, United States). These mice received a steady infusion of Klotho or vehicle starting at the time of implantation of the osmotic minipump. Mice were allowed to recover from surgical implantation for two days prior to exposure to SARS-CoV-2. Each vial of recombinant mouse Klotho (50 micrograms in 75 microliters) for the osmotic minipump treatment arms (cohorts 7 and 8) was diluted to 300 microliters in Klotho buffer. Each osmotic minipump for the five male and five female mice from cohorts 7 and 8 was loaded with 100 microliters of this diluted Klotho solution. The administered dose from the osmotic minipump was 0.05 mg/kg/day (per mouse) over the duration of the study. However, the infusion rate of each osmotic minipump was 0.25 microliters per hour, meaning a total dose of 6 microliters per day and 84 microliters for a maximum of 14 days (Day 12). Therefore, each osmotic minipump contained an absolute total of 16.7 micrograms of recombinant mouse Klotho protein in the 100 microliters of Klotho buffer in each minipump. Control cohorts 3 and 4 received 100 microliters of buffer solution only in each minipump.

### 2.5. Mice Receiving Intraperitoneal Injections

On day -2, all mice from cohorts 1, 2, 5, and 6 started to receive intraperitoneal (i.p.) injections. Each vial of mouse Klotho (50 micrograms in 75 microliters) for the treatment arms of the i.p. group (cohorts 5 and 6) was diluted up to a final volume of 10 mL with buffer solution such that 100 microliters contained 0.5 micrograms of Klotho. This Klotho solution was aliquoted into 1 mL tubes and frozen such that each 1 mL tube was thawed just prior to injection. Therefore, on day -2, five male and five female mice from cohorts 5 and 6 started to receive i.p. injections of mouse Klotho at a dose of 0.025 mg/kg twice a day (every 12 h) via i.p. injection (0.05 mg of Klotho per kg of bodyweight per day). Each injection contained 100 microliters of Klotho solution. The ten mice in the control cohorts (cohorts 1 and 2) from the i.p. group received 100 microliters of phosphate buffer solution in each i.p. injection (twice a day). Mice were anesthetized prior to handling for each i.p. injection.

On day 0, all mice in all eight cohorts were challenged with the SARS-CoV-2 viral load through nasal delivery. All mice were monitored and evaluated until death. Mice had free access to water and rodent chow. The health of each mouse was assessed on a daily basis by scoring health parameters included in the Animal Study Clinical Monitoring Chart.

Although each experiment was performed once, the second experiment included cohorts testing similar variables for recombinant mouse Klotho supplementation through i.p. injections to assess consistency of the first survival results.

We have provided the Health Charts of the experiments in the Supplementary Section.

### 2.6. Statistical Methods

Due to small sample size, a non-parametric assessment (Kruskal–Wallis test) was applied to compare the weight and temperature of the mice in each group (control, mouse Klotho, human Klotho) in the first experiment. Due to technical constraints (see Materials and Methods for the second animal study), we were not able to make such a comparison in the second experiment.

Non-parametric, semi-parametric, and parametric methods were used to analyze the survival data. We first applied the non-parametric log rank test to the data in order to test whether the survival curves were different. In addition, the p-value of the trend (p_trend_) was determined when comparing three or more survival curves. We applied semi-parametric Cox regression models and tested the proportional hazard assumption through the Schoenfeld residuals test. The Akaike Information Criterion and the Cox–Snell residuals plots were used to determine whether a parametric model fit the data better than the Cox model; if the model fits the data, then these residuals should have a standard exponential distribution with λ = 1. One way to verify the fit is to calculate an empirical estimate of the cumulative hazard function based on the Kaplan–Meier survival estimates or the Aalen–Nelson estimator, taking the Cox–Snell residuals as the time variable and the censoring variable as before and plotting it against Cox–Snell. If the model fits the data, the plot should be a straight line with a slope of 1. We then applied the optimal parametric model to generate hazard ratios (HR) and 95% confidence intervals. Parametric models for the first experiment were further adjusted for baseline weight and temperature. Models for the second experiment were sex-adjusted, and we additionally performed a sex-stratified analysis.

A meta-analysis of the hazard ratios from both studies was performed. As the heterogeneity (I^2^) was 0% for both the Cox and parametric model meta-analyses, we applied fixed effects to pool the data.

The parametric model that best fit the data for both experiments was the Weibull model.

SPSS, Stata version 15.1, and Comprehensive Meta-Analysis were used for statistical analysis. The Stata computer code is available on request.

## 3. Results

### 3.1. General Description

The baseline characteristics of the mice in the first experiment are displayed in Table 3a. The cohorts were defined by mouse Klotho, human Klotho, and vehicle. All mice were eight weeks old. There were no statistical differences in weight or temperature variables according to cohort group (Table 3b).

### 3.2. Survival Analysis: Kaplan–Meier

**Figure 1:**
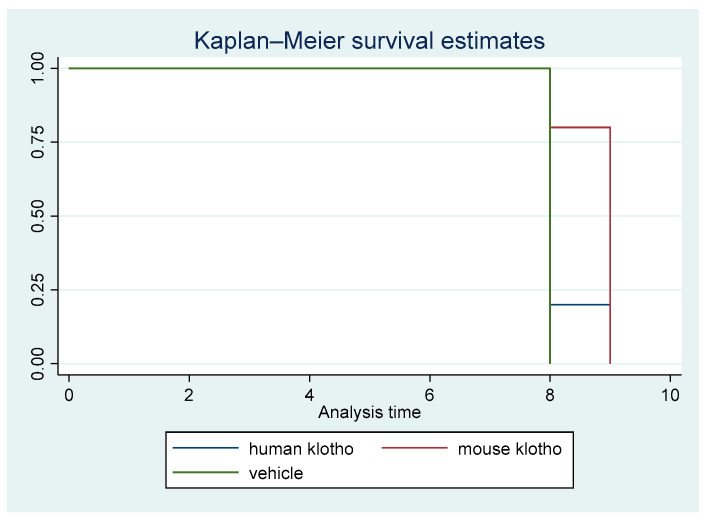
Kaplan–Meier survival curves for treatment with mouse Klotho versus human Klotho versus vehicle (all female mice). Log rank test *p* = 0.026; *p*_trend_ = 0.00.

A non-parametric survival analysis was carried out. Figure 1 displays the Kaplan–Meier survival curves for the first animal study. The difference in survival among the three cohorts was statistically significant (*p =* 0.026).

There was a clear trend in survival benefit for mice that received supplemental recombinant mouse Klotho versus the controls, whereas the trend in mice receiving recombinant human Klotho was weak, indicating protein sequence specificity for the beneficial effects of Klotho supplementation. The trend of increased survival in mice with mouse Klotho versus human Klotho versus vehicle was significant (*p*_trend_ = 0.009). This result was driven by a significant difference between the mouse Klotho and control groups (*p =* 0.014).

**Figure 2:**
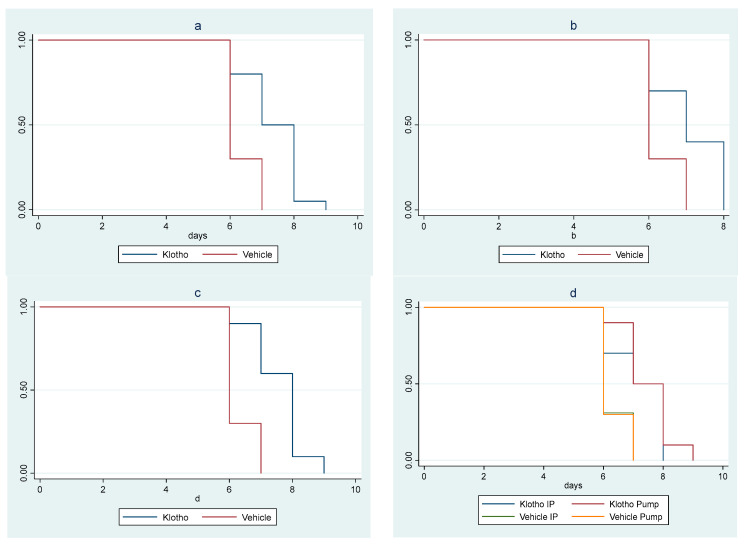
Kaplan–Meier survival curves for experiment with eight cohorts: (**a**) Klotho treatment versus vehicle, with sex and mode of treatment cohorts combined (log rank test *p* < 0.001); (**b**) Klotho treatment versus vehicle in male mice (mode of treatment combined, log rank test *p* = 0.019); (**c**) Klotho treatment versus vehicle in female mice (mode of treatment combined, log rank *p* = 0.01); (**d**) minipump versus Klotho in i.p. injections versus vehicle (sexes combined, log rank test *p* < 0.001, *p*_trend_ < 0.001).

In the second animal study, there was a significant survival difference among cohorts (*p* = 0.010). The trend of increasing survival across cohorts, from the control male mice cohort (vehicle i.p. method) to the female mice cohort receiving Klotho through osmotic minipump, was highly significant (*p*_trend_ < 0.001). A significant increase in survival in the Klotho group versus controls (*p* < 0.001) was observed when the cohorts were clustered by sex and delivery mode. Statistical significance was maintained when the analysis was sex-stratified (male mice *p* = 0.019; female mice *p* = 0.001) (Figure 2a–c).

Klotho treatment delivered by minipump provided a greater survival benefit than i.p. delivery of Klotho, which in turn provided a greater survival benefit than controls (*p* < 0.001; *p*_trend_ < 0.001) (Figure 2d).

### 3.3. Survival Analyses: Cox and Weibull Models

Concerning parametric survival analyses, we applied Weibull regression models (the best fit for our data; see Section 2). In the first experiment, Klotho treatment was associated with an unadjusted hazard ratio (HR) of 0.07 (0.01–0.44), *p* = 0.004, indicating that Klotho treatment decreased the rate of death by 93% compared to control. Repeating the Weibull model after adjusting for baseline temperature and weight did not significantly change the results (data not shown, available on request). In the second animal study, Klotho treatment was associated with a sex-adjusted HR of 0.13 (0.06–0.29), *p* < 0.001, indicating that Klotho treatment decreased the rate of mortality by 87% compared to control. When the analysis was stratified by sex, Klotho was associated with an HR of 0.18 (0.06–0.53), *p* = 0.002, n = 20 in male mice and an HR of 0.08 (0.02–0.28), *p* < 0.001, n = 20 in female mice.

A meta-analysis of the two studies showed that Klotho was associated with a pooled HR of 0.12 (0.06–0.25), *p* < 0.001 (exact *p* = 2.7 × 10^−8^). Overall, Klotho treatment decreased the rate of mortality by 88% compared to control. We found no evidence of heterogeneity (I^2^ = 0%).

The application of Cox models to the data resulted in an unadjusted HR for Klotho treatment of 0.50 (0.14–1.73), *p* = 0.273 in the first animal study and a sex-adjusted HR of 0.33 (0.15–0.71), *p* = 0.005 in the second animal study. The meta-analysis showed a pooled HR of 0.37 (0.19–0.72), *p* = 0.003. Again, there was no evidence of heterogeneity (I^2^ = 0%). The proportional hazard assumption was met in all cases. The adjustment of the Cox model of the first experiment by weight and temperature variables did not significantly change the results, similar to the parametric assessments.

## 4. Discussion

Klotho treatment or *Klotho* overexpression have been shown to provide health benefits using animal models in certain other experimental settings, such as acute to chronic kidney injury progression [58], cognitive impairment [42,43,46], and sepsis [44]. The experimental evidence demonstrates a statistically significant health benefit from Klotho supplementation in a COVID-19 animal model and strongly suggests that Klotho plays an important role in the disease pathogenesis induced by SARS-CoV-2 infection.

Our non-parametric survival analysis showed an increased survival benefit for animals that received exogenous recombinant Klotho supplementation. In addition, parametric Weibull models revealed a statistically significant survival benefit from Klotho supplementation in both male and female COVID-19 model mice, despite the small sample size of the cohorts. These research findings should attract more attention towards the possible interplay between the pathogenesis from infection by SARS-CoV-2 (as well as other coronaviruses, potentially) and the anti-aging protein Klotho.

Mechanistically, we hypothesize that Klotho supplementation may act via inhibition of the NF-κβ pathway [15], which is known to be activated by at least five different SARS-CoV-2 proteins [32]. NF-κβ signaling is a potent inductor of pro-inflammatory cytokines and chemokines, and in general is considered a central mediator of inflammasome activation [26,59].

This initial research deserves further investigation to address its limitations. as our cohort sample sizes were small, larger cohort sizes can be expected to further strengthen the statistical significance of the results. Dose–response studies, Klotho quantification and stability assays, evaluation of different signaling pathways, and the determination of Klotho distribution through animal organs were beyond the scope of this study, but would provide valuable insights into the possible mode of action of Klotho in COVID-19 pathogenesis as well as possible therapeutic targets. This preclinical study should be followed by studies evaluating Klotho levels in COVID-19 patients with varying classifications of disease severity. While ongoing clinical studies are currently evaluating the association of Klotho with different diseases, we have yet to identify any related to COVID-19 disease, much less a study evaluating a therapeutic intervention with Klotho.

## 5. Conclusions

Klotho supplementation was able to significantly increase survival in two independent studies of COVID-19 mice models after exposure to SARS-CoV-2. A meta-analysis reflected high consistency across cohorts, reinforcing this conclusion. We submit the premise that Klotho plays a central role in COVID-19 pathogenesis and that its acute deficit sharply increases the risk of case severity. The potential benefit from the administration of exogenous Klotho in the context of infections from other known coronaviruses, such as MERS and SARS, should be evaluated in animal models, as the potential development of a therapeutic applicable to coronaviruses in general would be highly desirable for future pandemic preparedness. The potential modulating effect of Klotho on “long COVID” should be investigated as well, in light of the burden of morbidity of its sequelae.

## Figures and Tables

**Table 1:** Correlation between Klotho levels or expression and risk factors for severity and lethality in COVID-19.

Risk Factor	Role or Correlation of Klotho	Ref.
Advanced age	Low Klotho is associated with shorter lifespan Serum Klotho decreases substantially with aging Aging is the strongest risk factor in COVID-19	[4,37]
Chronic kidney disease (CKD)	Main cause of systemic pan-Klotho deficiency Most potent risk factor for COVID-19 mortality after advanced age and cancer Klotho deficiency worsens CKD progression and induces the premature aging phenotype of CKD	[3,4,6,7,12]
Diabetes mellitus (DM)	*KL* is downregulated in type 2 DM DM (especially uncontrolled DM) is a risk factor for COVID-19 mortality	[4,35]
Obesity	Serum Klotho is decreased in obesity Obesity is a risk factor for COVID-19 mortality	[4,38]
Cancer	Klotho has been identified as a tumor suppressor through modulation of IGF-1 Cancer is a strong risk factor for COVID-19 mortality	[4,36]

Abbreviations: CKD: chronic kidney disease. KL: Klotho gene. DM: diabetes mellitus. IGF-1: insulin like growth factor 1.

**Table 2:** Correlation between Klotho levels or expression and clinical complications similar to those found in COVID-19 cases.

Complication	Role or Correlation of Klotho	Ref.
Acute kidney injury (AKI)	AKI induces a dramatic decrease in systemic Klotho levels and a status of pan-Klotho deficiency AKI is one of the most serious complications associated with mortality in COVID-19 The viral tropism in kidney is 100-fold greater than in lung tissue	[5,39,47]
Acute respiratory distress syndrome (lung-kidney axis)	AKI has a strong temporal association with respiratory failure and mechanical ventilation Klotho administration alleviates lung injury induced by kidney injury in animal models Klotho is a strong causal candidate underlying the lung-kidney axis, which is described in COVID-19 disease	[40,41,48]
(Micro) thrombosis	PAI-1 levels, a key molecule in thrombosis, are strikingly elevated in *Kl* deficient mice PAI-1 contributes to the aging phenotype as an important mediator of senescence COVID-19 induces a prothrombotic state according to meta-analysis	[49,50,51]
Cytokine release syndrome	IL-6 plays a key role in cytokine release syndrome Klotho downregulates endothelial IL-6 expression Klotho alleviates inflammation via modulation of Wnt1/pCREB pathway SARS-CoV-2 infection can induce cytokine release syndrome	[52,53]
Cognitive disorder	Klotho depletion in the choroid plexus induces inflammation and immune-mediated neuropathogenesis *Kl* overexpression enhances cognition in mice and nonhuman primates Klotho depletion impairs memory COVID-19 is associated with impairment in executive functioning	[42,43,46,54,55]
Multi-organ failure	Sepsis creates a state of Klotho deficiency in ICU patients, especially in the context of AKI Low Klotho levels correlate with major adverse kidney events in humans Pretreatment with recombinant Klotho alleviates organ damage and inflammation in rodent models with endotoxemia	[44,56,57]

Abbreviations: AKI: acute kidney injury. PAI-1. PAI-1: plasminogen activator inhibitor 1. IL-6: interleukin-6.

**Table 3:** Baseline characteristics of female mice in the first animal study (n = 15) and **b.** non-parametric comparisons of weight and temperature data across cohort groups in the first experiment.

a				
Mouse Number	Group	Baseline Age	Baseline Temperature	Baseline Weight
1	Control	8 weeks	37.4	19.5
2	Control	8 weeks	37.2	19
3	Control	8 weeks	37.4	20.5
4	Control	8 weeks	37.6	20
5	Control	8 weeks	36.9	19.5
6	Mouse Klotho	8 weeks	37.1	20
7	Mouse Klotho	8 weeks	38.1	21.5
8	Mouse Klotho	8 weeks	37.3	21.5
9	Mouse Klotho	8 weeks	37.2	19.5
10	Mouse Klotho	8 weeks	37.4	18.5
11	Human Klotho	8 weeks	37.4	18
12	Human Klotho	8 weeks	37.4	19.5
13	Human Klotho	8 weeks	37.8	21.5
14	Human Klotho	8 weeks	37.2	19.5
15	Human Klotho	8 weeks	37.0	19.5
**b**				
**Variable**			**Kruskal Wallis *p* value**	
Weight			0.645	
Temperature			0.989	

## Data Availability

All original data are available in the Supplementary Section as health charts.

## Supplementary Materials

The following supporting information can be downloaded at: https://www.mdpi.com/article/10.3390/pathogens12121404/s1, Health charts.
