# Supplementary material for: Exogenous Klotho Extends Survival in COVID-19 Model Mice
Source: Pathogens. 2023 Nov 29;12(12):1404. doi: 10.3390/pathogens12121404 (PMC10746004; doi:10.3390/pathogens12121404)
Supplement: Supplementary file 1 [file pathogens-12-01404-s001.zip › pathogens-2693784-supplementary.pdf]

Supplementary Materials for

Exogenous Klotho extends survival in COVID-19 model mice

Authors: Farhang Alem[1], Natalia Campos-Obando[2†], Aarthi Narayanan[1], Charles L. Bailey[1†], Roman F. Macaya[3,*]

[1] Biomedical Research Laboratory, George Mason University, 4400 University Dr, Fairfax, VA 22030, USA; falem@gmu.edu (F.A.); anaraya1@gmu.edu (A.N.); cbailey2@gmu.edu (C.L.B.)
[2] Formerly at Caja Costarricense de Seguro Social PO BOX: 10105-1000, San José, Costa Rica; n.camposobando@erasmusmc.nl
[3] Department of Global Health and Population, Harvard T.H. Chan School of Public Health, 665 Huntington Ave., Boston, MA 02115, USA
* Correspondence: rmacaya@hsph.harvard.edu
† Currently retired.

This file includes Health Charts

ORIGINAL DATA: HEALTH CHARTS

First animal study: Vehicle cohort, 5 female mice

**Protocol:**	**399**	**Species:**	**AC70 Mice**	**Building:**			
**Investigator:**		**Agent Administered:**	**SARS-CoV-2**	**Room Number:**		**CAGE #:**	**Control**
**Study Start:**	**8/11/21**	**Total # of Animals in cohort:**	**5**				

ANIMAL STUDY CLINICAL MONITORING CHART

Appearance	Mobility/Attitude	Signs of COVID-19	Body Condition	TOTAL SCORE
0 - Smooth coat, bright eyes	0 - Active/Scurrying, alert	0 - No respiratory distress	0 - Obese or Normal	0-4 = Normal 1x daily monitoring
1 - Slightly scruffy and/or hunched at rest	1 - Walking/Mild lethargic	1 - Decreased eating, discharge around eyes and nose	1 - Underconditioned	4-9 = 2x daily monitoring
2 - Scruffy and/or hunched at rest	2 - Slow movement/Lethargic	2 - Lethargic, wheezing, coughing, weight loss	2 - Emaciated	≥10 = Euthanize
3 - Very scruffy and/or hunched, Closed eyes	3 - No movement/ unresponsive	3 - Unaware, severe respiratory distress		FD = Found Dead
		Moist feed & feed on floor given post-infection		S or E = sacrificed/euthanized per study design

WHEN ADDITIONAL MONITORING IS REQUIRED, THE TOTAL NUMBER OF ANIMALS REMAINING IN THE CAGE WILL BE RECORDED DURING THE PM CHECK

UPON EUTHANIZING OR FINDING A DECEASED ANIMAL TRANSFER THE CARCASS TO THE DESIGNATED CARCASS FREEZER

Date	Time	Appearance					Mobility					Attitude					Body Condition					TOTAL SCORE					# of mice remaining during PM check	Initials
Animal ID →		0	1	2	3	4	0	1	2	3	4	0	1	2	3	4	0	1	2	3	4	**0**	**1**	**2**	**3**	**4**		
Day 0	AM																					**0**	**0**	**0**	**0**	**0**	5	FA
	PM																											

Day 1	AM																					**0**	**0**	**0**	**0**	**0**	5	FA
	PM																											
Day 2	AM																					**0**	**0**	**0**	**0**	**0**	5	FA
	PM																											
Day 3	AM																					**0**	**0**	**0**	**0**	**0**	5	FA
	PM																											
Day 4	AM																					**0**	**0**	**0**	**0**	**0**	5	FA
	PM																											
Day 5	AM																					**0**	**0**	**0**	**0**	**0**	5	FA
	PM																											
Day 6	AM																					**0**	**0**	**0**	**0**	**0**	5	FA
	PM																											
Day 7	AM																										5	FA
	PM	1	1	2	1	1	1	1	1	1	1						1			1		**3**	**2**	**3**	**3**	**2**		
Day 8	AM																										0	FA
	PM	3	3	3	3	3	3	3	3	3	3	3	3	3	3	3	2	2	2	2	2	**11**	**11**	**11**	**11**	**11**		
Day 9	AM																											
	PM																											

First animal study: human rKlotho dose, 5 female mice

Protocol:	399	Species:	AC70 Mice	Building:			
Investigator:		Agent Administered:	SARS-CoV-2	Room Number:		CAGE #:	Human rKlotho
Study Start:	8/11/21	Total # of Animals in cohort:	5				

ANIMAL STUDY CLINICAL MONITORING CHART

Appearance	Mobility/Attitude	Signs of COVID-19	Body Condition	TOTAL SCORE
0 - Smooth coat, bright eyes	0 - Active/Scurrying, alert	0 - No respiratory distress	0 - Obese or Normal	0-4 = Normal 1x daily monitoring
1 - Slightly scruffy and/or hunched at rest	1 - Walking/Mild lethargic	1 - Decreased eating, discharge around eyes and nose	1 - Underconditioned	4-9 = 2x daily monitoring
2 - Scruffy and/or hunched at rest	2 - Slow movement/Lethargic	2 - Lethargic, wheezing, coughing, weight loss	2 - Emaciated	≥10 = Euthanize
3 - Very scruffy and/or hunched, Closed eyes	3 - No movement/ unresponsive	3 - Unaware, severe respiratory distress		FD = Found Dead
		Moist feed & feed on floor given post-infection		S or E = sacrificed/euthanized per study design

WHEN ADDITIONAL MONITORING IS REQUIRED, THE TOTAL NUMBER OF ANIMALS REMAINING IN THE CAGE WILL BE RECORDED DURING THE PM CHECK

UPON EUTHANIZING OR FINDING A DECEASED ANIMAL TRANSFER THE CARCASS TO THE DESIGNATED CARCASS FREEZER

Date	Time	Appearance					Mobility					Attitude					Body Condition					TOTAL SCORE					# of mice remaining during PM check	Initials
Animal ID →		0	1	2	3	4	0	1	2	3	4	0	1	2	3	4	0	1	2	3	4	**0**	**1**	**2**	**3**	**4**		
	AM																					**0**	**0**	**0**	**0**	**0**	5	FA

Day 0	PM																											
Day 1	AM																					**0**	**0**	**0**	**0**	**0**	5	FA
	PM																											
Day 2	AM																					**0**	**0**	**0**	**0**	**0**	5	FA
	PM																											
Day 3	AM																					**0**	**0**	**0**	**0**	**0**	5	FA
	PM																											
Day 4	AM																					**0**	**0**	**0**	**0**	**0**	5	FA
	PM																											
Day 5	AM																					**0**	**0**	**0**	**0**	**0**	5	FA
	PM																											
Day 6	AM																					**0**	**0**	**0**	**0**	**0**	5	FA
	PM																											
Day 7	AM																										5	FA
	PM	1		1	1	1	1		1	1	1					1	1					**3**		**2**	**2**	**3**		
Day 8	AM																										1	FA
	PM		2					1					1					1					**5**					
Day 9	AM																										0	FA
	PM		3					3					3					2					**1**<br>**1**					
Day 10	AM																											
	PM																											

First animal study: mouse rKlotho dose, 5 female mice

**Protocol:**	**399**	**Species:**	**AC70 Mice**	**Building:**			
**Investigator:**		**Agent Administered:**	**SARS-CoV-2**	**Room Number:**		**CAGE #:**	**Mouse rKlotho**
**Study Start:**	**8/11/21**	**Total # of Animals in cohort:**	**5**				

ANIMAL STUDY CLINICAL MONITORING CHART

Appearance	Mobility/Attitude	Signs of COVID-19	Body Condition	TOTAL SCORE
0 - Smooth coat, bright eyes	0 - Active/Scurrying, alert	0 - No respiratory distress	0 - Obese or Normal	0-4 = Normal 1x daily monitoring
1 - Slightly scruffy and/or hunched at rest	1 - Walking/Mild lethargic	1 - Decreased eating, discharge around eyes and nose	1 - Underconditioned	4-9 = 2x daily monitoring
2 - Scruffy and/or hunched at rest	2 - Slow movement/Lethargic	2 - Lethargic, wheezing, coughing, weight loss	2 - Emaciated	≥10 = Euthanize
3 - Very scruffy and/or hunched, Closed eyes	3 - No movement/ unresponsive	3 - Unaware, severe respiratory distress		FD = Found Dead
		Moist feed & feed on floor given post-infection		S or E = sacrificed/euthanized per study design

WHEN ADDITIONAL MONITORING IS REQUIRED, THE TOTAL NUMBER OF ANIMALS REMAINING IN THE CAGE WILL BE RECORDED DURING THE PM CHECK

UPON EUTHANIZING OR FINDING A DECEASED ANIMAL TRANSFER THE CARCASS TO THE DESIGNATED CARCASS FREEZER

Date	Time	Appearance					Mobility					Attitude					Body Condition					TOTAL SCORE					# of mice remaining during PM check	Initials
Animal ID →		0	1	2	3	4	0	1	2	3	4	0	1	2	3	4	0	1	2	3	4	0	1	2	3	4		
	AM																					0	0	0	0	0	5	FA

Day 0	PM																											
Day 1	AM																					0	0	0	0	0	5	FA
	PM																											
Day 2	AM																					0	0	0	0	0	5	FA
	PM																											
Day 3	AM																					0	0	0	0	0	5	FA
	PM																											
Day 4	AM																					0	0	0	0	0	5	FA
	PM																											
Day 5	AM																					0	0	0	0	0	5	FA
	PM																											
Day 6	AM																					0	0	0	0	0	5	FA
	PM																											
Day 7	AM																					0	0	0	0	0	5	FA
	PM																											
Day 8	AM																										4	FA
	PM		1		1			1		2	2		1	1	1	1		1	1	1	1		4	1	5	4		
Day 9	AM																										0	FA
	PM		3	3	3	3		3	3	3	3		3	3	2	3		2	2	2	2		1<br>1	11	10	1<br>1		
Day 10	AM																											
	PM																											
Day 11	AM																											
	PM																											
Day 12	AM																											
	PM																											
Day 13	AM																											
	PM																											
	AM																											

Day 14	PM																											
Day 15	AM																											
	PM																											
Day 16	AM																											
	PM																											
Day 17	AM																											
	PM																											
Day 18	AM																											
	PM																											
Day 19	AM																											
	PM																											
Day 20	AM																											
	PM																											
Day 21	AM																											
	PM																											

Second animal study: vehicle male IP, 5 mice

**Protocol:**	**0399**	**Species:**	**AC 70 Mice**	**Building:**			
**Investigator:**		**Agent Administered:**	**SARS-CoV-2**	**Room Number:**		**CAGE #:**	**Vehicle-Male-IP**
**Study Start:**	**8/9/22**	**Total # of Animals in cohort:**	**5**				

ANIMAL STUDY CLINICAL MONITORING CHART

Appearance	Mobility/Attitude	Signs of COVID-19	Body Condition	TOTAL SCORE
0 - Smooth coat, bright eyes	0 - Active/Scurrying, alert	0 - No respiratory distress	0 - Obese or Normal	≥2 in any category or a total score of 0-5 = Normal 1x daily monitoring
1 - Slightly scruffy and/or hunched at rest	1 - Walking/Mild lethargic	1 - Decreased eating, discharge around eyes and nose	2 - Underconditioned	Score of 4 in any single category or a total score of ≥ 10 = Euthanize
2 - Scruffy and/or hunched at rest	2 - Slow movement/Lethargic	2 - Lethargic, wheezing, coughing, weight loss	4 - Emaciated	20% or greater weight loss = Euthanize
3 - Very scruffy and/or hunched, mild eye crust	3 - No movement/ Unaware	3 - Unresponsive to stimuli, difficulty breathing		FD = Found Dead
4 - Very scruffy and/or hunched, closed eyes	4 - Unresponsive	4. Unaware, severe respiratory distress		S or E = sacrificed/euthanized per study design

WHEN ADDITIONAL MONITORING IS REQUIRED, THE TOTAL NUMBER OF ANIMALS REMAINING IN THE CAGE WILL BE RECORDED DURING THE PM CHECK

UPON EUTHANIZING OR FINDING A DECEASED ANIMAL TRANSFER THE CARCASS TO THE DESIGNATED CARCASS FREEZER

Moist feed & feed on floor given post-infection

Date	Time	Appearance					Mobility					Attitude					Body Condition					TOTAL SCORE					# of mice remaining during PM check	Initials
Animal ID →		0	1	2	3	4	0	1	2	3	4	0	1	2	3	4	0	1	2	3	4	**0**	**1**	**2**	**3**	**4**		
Day 0	AM																										5	
	PM																											

Day 1	AM																										5	
	PM																											
Day 2	AM																										5	
	PM																											
Day 3	AM																										5	
	PM																											
Day 4	AM	2	1	2	1	0	2	0	2	1	0	1	0	1	0	0	0	0	0	0	0	**5**	**1**	**5**	**2**	**0**	5	
	PM																											
Day 5	AM	3	2	3	3	1	3	2	3	2	0	1	2	1	1	0	2	1	2	2	0	**9**	**7**	**9**	**8**	**1**	5	
	PM																											
Day 6	AM		2			1		2			2		1			1		2			2	**X**	**X**	**X**	**X**	**6**	1	
	PM																											
Day 7	AM		3			3		2			2		3			2		2			2					**X**	0	
	PM																											
Day 8	AM																										0	
	PM																											

Second animal study: Vehicle female IP, 5 mice

**Protocol:**	**0399**	**Species:**	**AC70 Mice**	**Building:**		**CAGE #:**	**Vehicle-Female-IP**
**Investigator:**		**Agent Administered:**	**SARS-CoV-2**	**Room Number:**			
**Study Start:**	**8/9/22**	**Total # of Animals in cohort:**	**5**				

ANIMAL STUDY CLINICAL MONITORING CHART

Appearance	Mobility/Attitude	Signs of COVID-19	Body Condition	TOTAL SCORE
0 - Smooth coat, bright eyes	0 - Active/Scurrying, alert	0 - No respiratory distress	0 - Obese or Normal	≥2 in any category or a total score of 0-5 = Normal 1x daily monitoring
1 - Slightly scruffy and/or hunched at rest	1 - Walking/Mild lethargic	1 - Decreased eating, discharge around eyes and nose	2 - Underconditioned	Score of 4 in any single category or a total score of ≥ 10 = Euthanize
2 - Scruffy and/or hunched at rest	2 - Slow movement/Lethargic	2 - Lethargic, wheezing, coughing, weight loss	4 - Emaciated	20% or greater weight loss = Euthanize
3 - Very scruffy and/or hunched, mild eye crust	3 - No movement/ Unaware	3 - Unresponsive to stimuli, difficulty breathing		FD = Found Dead
4 - Very scruffy and/or hunched, closed eyes	4 - Unresponsive	4. Unaware, severe respiratory distress		S or E = sacrificed/euthanized per study design

WHEN ADDITIONAL MONITORING IS REQUIRED, THE TOTAL NUMBER OF ANIMALS REMAINING IN THE CAGE WILL BE RECORDED DURING THE PM CHECK

UPON EUTHANIZING OR FINDING A DECEASED ANIMAL TRANSFER THE CARCASS TO THE DESIGNATED CARCASS FREEZER

Moist feed & feed on floor given post-infection

Date	Time	Appearance					Mobility					Attitude					Body Condition					TOTAL SCORE					# of mice remaining during PM check	Initials
Animal ID →		0	1	2	3	4	0	1	2	3	4	0	1	2	3	4	0	1	2	3	4	**0**	**1**	**2**	**3**	**4**		
Day 0	AM																										5	
	PM																											

Day 1	AM																										5	
	PM																											
Day 2	AM																										5	
	PM																											
Day 3	AM																										5	
	PM																											
Day 4	AM																										5	
	PM																											
Day 5	AM	1	2	1	1	0	1	1	1	1	0	1	1	1	1	0	0	0	0	0	0	**3**	**4**	**3**	**3**	**0**	5	
	PM																											
Day 6	AM	3	2	3	3	1	2	2	3	2	1	3	2	3	3	1	2	2	2	2	0	**10**	**8**	**11**	**10**	**3**	2	
	PM																											
Day 7	AM	X	4	X	X	3	X	3	X	X	3	X	3	X	X	3	X	2	X	X	4	**X**	**12**	**X**	**X**	**12**	0	
	PM																						**X**			**X**		
Day 8	AM																											
	PM																											

Second animal study: Klotho IP male mice, 5 mice

**Protocol:**	**0399**	**Species:**	**AC70 Mice**	**Building:**			
**Investigator:**		**Agent Administered:**	**SARS-CoV-2**	**Room Number:**		**CAGE #:**	**Klotho-Male-IP**
**Study Start:**	**8/9/22**	**Total # of Animals in cohort:**	**5**				

ANIMAL STUDY CLINICAL MONITORING CHART

Appearance	Mobility/Attitude	Signs of COVID-19	Body Condition	TOTAL SCORE
0 - Smooth coat, bright eyes	0 - Active/Scurrying, alert	0 - No respiratory distress	0 - Obese or Normal	≥2 in any category or a total score of 0-5 = Normal 1x daily monitoring
1 - Slightly scruffy and/or hunched at rest	1 - Walking/Mild lethargic	1 - Decreased eating, discharge around eyes and nose	2 - Underconditioned	Score of 4 in any single category or a total score of ≥ 10 = Euthanize
2 - Scruffy and/or hunched at rest	2 - Slow movement/Lethargic	2 - Lethargic, wheezing, coughing, weight loss	4 - Emaciated	20% or greater weight loss = Euthanize
3 - Very scruffy and/or hunched, mild eye crust	3 - No movement/ Unaware	3 - Unresponsive to stimuli, difficulty breathing		FD = Found Dead
4 - Very scruffy and/or hunched, closed eyes	4 - Unresponsive	4. Unaware, severe respiratory distress		S or E = sacrificed/euthanized per study design

WHEN ADDITIONAL MONITORING IS REQUIRED, THE TOTAL NUMBER OF ANIMALS REMAINING IN THE CAGE WILL BE RECORDED DURING THE PM CHECK

UPON EUTHANIZING OR FINDING A DECEASED ANIMAL TRANSFER THE CARCASS TO THE DESIGNATED CARCASS FREEZER

Moist feed & feed on floor given post-infection

Date	Time	Appearance					Mobility					Attitude					Body Condition					TOTAL SCORE					# of mice remaining during PM check	Initials
Animal ID →		0	1	2	3	4	0	1	2	3	4	0	1	2	3	4	0	1	2	3	4	**0**	**1**	**2**	**3**	**4**		
Day 0	AM																										5	
	PM																											
Day 1	AM																										5	

	PM																											
Day 2	AM																										5	
	PM																											
Day 3	AM																										5	
	PM																											
Day 4	AM	1	1	1	0	1	1	1	0	0	2	0	0	0	0	0	0	0	0	0	0	**2**	**2**	**1**	**0**	**3**	5	
	PM																											
Day 5	AM	0	3	3	1	0	0	2	2	1	0	0	2	2	1	0	0	2	2	0	0	**0**	**9**	**9**	**3**	**0**	5	
	PM																											
Day 6	AM	1	X	X	3	1	1	X	X	2	1	1	X	X	2	1	0	X	X	2	0	**3**	**X**	**X**	**9**	**3**	3	
	PM																											
Day 7	AM	3	X	X	X	2	2	X	X	X	2	2	X	X	X	2	2	X	X	X	2	**9**	**X**	**X**	**X**	**8**	2	
	PM																											
Day 8	AM	X	X	X	X	X	X	X	X	X	X	X	X	X	X	X	X	X	X	X	X	**X**	**X**	**X**	**X**	**X**	0	
	PM																											
Day 9	AM																											
	PM																											
Day 10	AM																											
	PM																											

Second animal study: Klotho IP female mice, 5 mice

Protocol:	0399	Species:	AC70 Mice	Building:		CAGE #:	Kotho-Female-IP
Investigator:		Agent Administered:	SARS-CoV-2	Room Number:			
Study Start:	8/9/22	Total # of Animals in cohort:	5				

ANIMAL STUDY CLINICAL MONITORING CHART

Appearance	Mobility/Attitude	Signs of COVID-19	Body Condition	TOTAL SCORE
0 - Smooth coat, bright eyes	0 - Active/Scurrying, alert	0 - No respiratory distress	0 - Obese or Normal	≥2 in any category or a total score of 0-5 = Normal 1x daily monitoring
1 - Slightly scruffy and/or hunched at rest	1 - Walking/Mild lethargic	1 - Decreased eating, discharge around eyes and nose	2 - Underconditioned	Score of 4 in any single category or a total score of ≥ 10 = Euthanize
2 - Scruffy and/or hunched at rest	2 - Slow movement/Lethargic	2 - Lethargic, wheezing, coughing, weight loss	4 - Emaciated	20% or greater weight loss = Euthanize
3 - Very scruffy and/or hunched, mild eye crust	3 - No movement/ Unaware	3 - Unresponsive to stimuli, difficulty breathing		FD = Found Dead
4 - Very scruffy and/or hunched, closed eyes	4 - Unresponsive	4. Unaware, severe respiratory distress		S or E = sacrificed/euthanized per study design

WHEN ADDITIONAL MONITORING IS REQUIRED, THE TOTAL NUMBER OF ANIMALS REMAINING IN THE CAGE WILL BE RECORDED DURING THE PM CHECK

UPON EUTHANIZING OR FINDING A DECEASED ANIMAL TRANSFER THE CARCASS TO THE DESIGNATED CARCASS FREEZER

Moist feed & feed on floor given post-infection

Date	Time	**Appearance**					**Mobility**					**Attitude**					**Body Condition**					TOTAL SCORE					# of mice remaining during PM check	Initials
Animal ID →		0	1	2	3	4	0	1	2	3	4	0	1	2	3	4	0	1	2	3	4	**0**	**1**	**2**	**3**	**4**		
Day 0	AM																										5	
	PM																											
Day 1	AM																										5	
	PM																											

Day 2	AM																									5	
	PM																										
Day 3	AM																									5	
	PM																										
Day 4	AM																									5	
	PM																										
Day 5	AM	1	1	2	1	2	1	2	1	1	1	0	1	0	0	0	0	0	0	0	0	**2**	**3**	**5**	**2**	**2**	5	
	PM																											
Day 6	AM	1	2	X	1	2	2	2	X	2	2	1	1	X	1	2	0	0	X	2	2	**4**	**5**	**X**	**6**	**8**	4	
	PM																											
Day 7	AM	3	2	X	2	X	2	2	X	3	X	2	3	X	2	X	2	2	X	4	X	**9**	**9**	**X**	**11**	**X**	3	
	PM																											
Day 8	AM	X	X	x	X	X	X	X	X	X	X	X	X	X	x	X	X	X	X	X	X	**X**	**X**	**X**	**X**	**X**	0	
	PM																											
Day 9	AM																											
	PM																											

Second animal study: Vehicle male pump, 5 mice

**Protocol:**	**0399**	**Species:**	**AC70 Mice**	**Building:**		**CAGE #:**	**Vehicle-Male-Pump**
**Investigator:**		**Agent Administered:**	**SARS-CoV-2**	**Room Number:**			
**Study Start:**	**8/9/22**	**Total # of Animals in cohort:**	**5**				

ANIMAL STUDY CLINICAL MONITORING CHART

Appearance	Mobility/Attitude	Signs of COVID-19	Body Condition	TOTAL SCORE
0 - Smooth coat, bright eyes	0 - Active/Scurrying, alert	0 - No respiratory distress	0 - Obese or Normal	≥2 in any category or a total score of 0-5 = Normal 1x daily monitoring
1 - Slightly scruffy and/or hunched at rest	1 - Walking/Mild lethargic	1 - Decreased eating, discharge around eyes and nose	2 - Underconditioned	Score of 4 in any single category or a total score of ≥ 10 = Euthanize
2 - Scruffy and/or hunched at rest	2 - Slow movement/Lethargic	2 - Lethargic, wheezing, coughing, weight loss	4 - Emaciated	20% or greater weight loss = Euthanize
3 - Very scruffy and/or hunched, mild eye crust	3 - No movement/ Unaware	3 - Unresponsive to stimuli, difficulty breathing		FD = Found Dead
4 - Very scruffy and/or hunched, closed eyes	4 - Unresponsive	4. Unaware, severe respiratory distress		S or E = sacrificed/euthanized per study design

WHEN ADDITIONAL MONITORING IS REQUIRED, THE TOTAL NUMBER OF ANIMALS REMAINING IN THE CAGE WILL BE RECORDED DURING THE PM CHECK

UPON EUTHANIZING OR FINDING A DECEASED ANIMAL TRANSFER THE CARCASS TO THE DESIGNATED CARCASS FREEZER

Moist feed & feed on floor given post-infection

Date	Time	**Appearance**					**Mobility**					**Attitude**					**Body Condition**					**TOTAL SCORE**					# of mice remaining during PM check	Initials
Animal ID →		0	1	2	3	4	0	1	2	3	4	0	1	2	3	4	0	1	2	3	4	**0**	**1**	**2**	**3**	**4**		
Day 0	AM																										5	
	PM																											
Day 1	AM																										5	
	PM																											

Day 2	AM																										5	
	PM																											
Day 3	AM																										5	
	PM																											
Day 4	AM	1	1	1	1	1	0	1	1	1	0	0	0	0	0	0	0	0	0	0	0	**1**	**2**	**2**	**2**	**1**	5	
	PM																											
Day 5	AM	1	3	3	1	1	1	1	2	2	1	1	2	2	2	0	0	2	2	2	0	**3**	**8**	**9**	**9**	**2**	5	
	PM																											
Day 6	AM	3	X	X	X	3	2	X	X	X	3	2	X	X	X	2	2	X	X	X	2	**9**	**X**	**X**	**X**	**10**	2	
	PM																											
Day 7	AM	X	X	X	X	X	X	X	X	X	X	X	X	X	X	X	X	X	X	X	X	**X**	**X**	**X**	**X**	**X**	0	
	PM																											
Day 8	AM																											
	PM																											

Second animal study: Vehicle Female Pump, 5 mice

**Protocol:**	**0399**	**Species:**	**AC70 Mice**	**Building:**		**CAGE #:**	**Vehicle-Female-Pump**
**Investigator:**		**Agent Administered:**	**SARS-CoV-2**	**Room Number:**			
**Study Start:**	**8/9/22**	**Total # of Animals in cohort:**	**5**				

ANIMAL STUDY CLINICAL MONITORING CHART

Appearance	Mobility/Attitude	Signs of COVID-19	Body Condition	TOTAL SCORE
0 - Smooth coat, bright eyes	0 - Active/Scurrying, alert	0 - No respiratory distress	0 - Obese or Normal	≥2 in any category or a total score of 0-5 = Normal 1x daily monitoring
1 - Slightly scruffy and/or hunched at rest	1 - Walking/Mild lethargic	1 - Decreased eating, discharge around eyes and nose	2 - Underconditioned	Score of 4 in any single category or a total score of ≥ 10 = Euthanize
2 - Scruffy and/or hunched at rest	2 - Slow movement/Lethargic	2 - Lethargic, wheezing, coughing, weight loss	4 - Emaciated	20% or greater weight loss = Euthanize
3 - Very scruffy and/or hunched, mild eye crust	3 - No movement/ Unaware	3 - Unresponsive to stimuli, difficulty breathing		FD = Found Dead
4 - Very scruffy and/or hunched, closed eyes	4 - Unresponsive	4. Unaware, severe respiratory distress		S or E = sacrificed/euthanized per study design

WHEN ADDITIONAL MONITORING IS REQUIRED, THE TOTAL NUMBER OF ANIMALS REMAINING IN THE CAGE WILL BE RECORDED DURING THE PM CHECK

UPON EUTHANIZING OR FINDING A DECEASED ANIMAL TRANSFER THE CARCASS TO THE DESIGNATED CARCASS FREEZER

Moist feed & feed on floor given post-infection

Date	Time	Appearance					Mobility					Attitude					Body Condition					TOTAL SCORE					# of mice remaining during PM check	Initials
Animal ID →		0	1	2	3	4	0	1	2	3	4	0	1	2	3	4	0	1	2	3	4	**0**	**1**	**2**	**3**	**4**		
Day 0	AM																										5	
	PM																											
Day 1	AM																										5	
	PM																											

Day 2	AM																										5	
	PM																											
Day 3	AM																										5	
	PM																											
Day 4	AM																										5	
	PM																											
Day 5	AM	3	3	3	3	1	2	3	2	2	1	2	2	2	2	1	2	2	2	2	0	**9**	**10**	**9**	**9**	**3**	5	
	PM																											
Day 6	AM	X	X	X	X	3	X	X	X	X	2	X	X	X	X	3	X	X	X	X	2	**X**	**X**	**X**	**X**	**10**	1	
	PM																											
Day 7	AM	X	X	X	X	X	X	X	X	X	X	X	X	X	X	X	X	X	X	X	X	**X**	**X**	**X**	**X**	**X**	0	
	PM																											
Day 8	AM																											
	PM																											

Second animal study: Klotho male pump, 5 mice

**Protocol:**	**0399**	**Species:**	**AC70 Mice**	**Building:**		**CAGE #:**	**Klotho-Male-Pump**
**Investigator:**		**Agent Administered:**	**SARS-CoV-2**	**Room Number:**			
**Study Start:**	**8/9/22**	**Total # of Animals in cohort:**	**5**				

ANIMAL STUDY CLINICAL MONITORING CHART

Appearance	Mobility/Attitude	Signs of COVID-19	Body Condition	TOTAL SCORE
0 - Smooth coat, bright eyes	0 - Active/Scurrying, alert	0 - No respiratory distress	0 - Obese or Normal	≥2 in any category or a total score of 0-5 = Normal 1x daily monitoring
1 - Slightly scruffy and/or hunched at rest	1 - Walking/Mild lethargic	1 - Decreased eating, discharge around eyes and nose	2 - Underconditioned	Score of 4 in any single category or a total score of ≥ 10 = Euthanize
2 - Scruffy and/or hunched at rest	2 - Slow movement/Lethargic	2 - Lethargic, wheezing, coughing, weight loss	4 - Emaciated	20% or greater weight loss = Euthanize
3 - Very scruffy and/or hunched, mild eye crust	3 - No movement/ Unaware	3 - Unresponsive to stimuli, difficulty breathing		FD = Found Dead
4 - Very scruffy and/or hunched, closed eyes	4 - Unresponsive	4. Unaware, severe respiratory distress		S or E = sacrificed/euthanized per study design

WHEN ADDITIONAL MONITORING IS REQUIRED, THE TOTAL NUMBER OF ANIMALS REMAINING IN THE CAGE WILL BE RECORDED DURING THE PM CHECK

UPON EUTHANIZING OR FINDING A DECEASED ANIMAL TRANSFER THE CARCASS TO THE DESIGNATED CARCASS FREEZER

Moist feed & feed on floor given post-infection

Date	Time	**Appearance**					**Mobility**					**Attitude**					**Body Condition**					**TOTAL SCORE**					# of mice remaining during PM check	Initials
Animal ID →		0	1	2	3	4	0	1	2	3	4	0	1	2	3	4	0	1	2	3	4	**0**	**1**	**2**	**3**	**4**		
Day 0	AM																										5	
	PM																											
Day 1	AM																										5	
	PM																											
Day 2	AM																										5	

	PM																											
Day 3	AM																										5	
	PM																											
Day 4	AM																										5	
	PM																											
Day 5	AM	1	1	1	3	1	0	0	0	2	0	0	0	0	2	0	0	0	0	2	0	**1**	**1**	**1**	**9**	**1**	5	
	PM																											
Day 6	AM	2	2	2	X	2	2	2	1	X	2	1	2	1	X	1	0	2	2	X	0	**5**	**8**	**6**	**X**	**5**	4	
	PM																											
Day 7	AM	X	X	3	X	3	X	X	3	X	3	X	X	2	X	2	X	X	2	X	2	**X**	**X**	**10**	**X**	**10**	2	
	PM																											
Day 8	AM	X	X	X	X	X	X	X	X	X	X	X	X	X	X	X	X	X	X	X	X	**X**	**X**	**X**	**X**	**X**	0	
	PM																											
Day 9	AM																											
	PM																											

Second animal study: Klotho female pump, 5 mice

**Protocol:**	**0399**	**Species:**	**AC70 Mice**	**Building:**		**CAGE #:**	**Klotho-Female-Pump**
**Investigator:**		**Agent Administered:**	**SARS-CoV-2**	**Room Number:**			
**Study Start:**	**8/9/22**	**Total # of Animals in cohort:**	**5**				

ANIMAL STUDY CLINICAL MONITORING CHART

Appearance	Mobility/Attitude	Signs of COVID-19	Body Condition	TOTAL SCORE
0 - Smooth coat, bright eyes	0 - Active/Scurrying, alert	0 - No respiratory distress	0 - Obese or Normal	≥2 in any category or a total score of 0-5 = Normal 1x daily monitoring
1 - Slightly scruffy and/or hunched at rest	1 - Walking/Mild lethargic	1 - Decreased eating, discharge around eyes and nose	2 - Underconditioned	Score of 4 in any single category or a total score of ≥ 10 = Euthanize
2 - Scruffy and/or hunched at rest	2 - Slow movement/Lethargic	2 - Lethargic, wheezing, coughing, weight loss	4 - Emaciated	20% or greater weight loss = Euthanize
3 - Very scruffy and/or hunched, mild eye crust	3 - No movement/ Unaware	3 - unresponsive to stimuli, difficulty breathing		FD = Found Dead
4 - Very scruffy and/or hunched, closed eyes	4 - Unresponsive	4. Unaware, severe respiratory distress		S or E = sacrificed/euthanized per study design

WHEN ADDITIONAL MONITORING IS REQUIRED, THE TOTAL NUMBER OF ANIMALS REMAINING IN THE CAGE WILL BE RECORDED DURING THE PM CHECK

UPON EUTHANIZING OR FINDING A DECEASED ANIMAL TRANSFER THE CARCASS TO THE DESIGNATED CARCASS FREEZER

Moist feed & feed on floor given post-infection

Date	Time	Appearance					Mobility					Attitude					Body Condition					TOTAL SCORE					# of mice remaining during PM check	Initials
Animal ID →		0	1	2	3	4	0	1	2	3	4	0	1	2	3	4	0	1	2	3	4	0	1	2	3	4		
Day 0	AM																										5	
	PM																											
Day 1	AM																										5	
	PM																											

Day 2	AM																										5	
	PM																											
Day 3	AM																										5	
	PM																											
Day 4	AM																										5	
	PM																											
Day 5	AM	0	0	1	1	1	0	0	1	1	0	0	0	0	0	0	0	0	0	0	0	**0**	**0**	**2**	**2**	**1**	5	
	PM																											
Day 6	AM	2	2	1	3	3	2	2	1	3	3	1	0	0	1	0	0	0	0	2	2	**5**	**4**	**2**	**9**	**8**	5	
	PM																											
Day 7	AM	3	3	2	X	X	3	3	2	X	X	1	2	2	X	X	2	2	X	X	X	**8**	**9**	**6**	**X**	**X**	3	
	PM																											
Day 8	AM	X	X	3	X	X	X	X	3	X	X	X	X	2	X	X	X	X	2	X	X	**X**	**X**	**10**	**X**	**X**	1	
	PM																											
Day 9	AM	X	X	X	X	X	X	X	X	X	x	X	X	X	X	X	X	X	X	X	X	**X**	**X**	**X**	**X**	**X**	0	
	PM																											
